# Case of a Painful Affection of the Arm, Following Venesection, Cured by Acupuncturation

**Published:** 1825-07

**Authors:** J. Webster


**Art. VI.**—***Case of a painful Affection of the Arm, following Venesection, cured by Acupuncturation.*** **By J. Webster, M.D.**

In compliance with your request, I send an account of the case in which acupuncturation was performed with the most decided benefit. It is the only instance wherein I have had recourse to the operation; but, while such opposite opinions exist with regard to the utility of the remedy, even one proof of its efficacy may not be altogether without interest.

10th February, 1825.—Mrs. Good, æt. thirty-two years, married, and in the sixth month of pregnancy, became a patient at the St. George's and St. James's Dispensary. On admission, it was stated that, about four months previously, she had been bled in the right arm, in consequence of a pain in the head, accompanied with giddiness. At the moment the vein was opened, she felt a most excruciating pain, very different from what she had previously experienced from the operation. Whilst the blood was flowing, she fainted, and continued in a swooning state for nearly an hour. The wound healed kindly, and during a week afterwards she was free from complaint. About the end of that time, she suddenly experienced a severe pain, proceeding from the bend of the elbow, and extending down the inner side of the right forearm, in the course of the inner cutaneous nerve, to the hand; the two fore-fingers and thumb were also similarly affected. This pain was most severely felt about two inches below the inner condyl of the humerus, and in the muscles composing the fleshy part of the thumb. There was also a prickling sensation at the points of the fingers; and she complained of a pain situated near the spinous process of the right scapula. "These symptoms," it is mentioned in the notes of the case, "have continued ever since, and have gradually increased in severity. At present, the pain is more excruciating than ever, especially in the upper part of the forearm and in the cicatrix of the vein, but it never extends above this point; nor has there been any swelling or puffiness of the limb, of which she has now almost lost the use. She feels always worse during the night, particularly when warm in bed. Was never affected with rheumatism, and her general health is good, although the tongue is slightly furred, and the bowels are a little costive."

Before admission, some aperient medicines had been prescribed, also a blister to the shoulder; and pieces of flannel, soaked in spirits, had been applied to the forearm; but from neither of these did she receive any benefit.

10th March.—During the last month has attended regularly at the Dispensary, and has taken aloetic purges, blue-pill, and colocynth; also saline medicines, camphor, valerian, and assafœtida. Volatile alkali, camphor, and soap liniments, with tincture of opium, have been rubbed upon the forearm, and hot fomentations have been applied; but all without the slightest effect in diminishing the pain. The only remedy that appeared to afford relief was a large blister, which extended from the bend of the arm to the wrist: whilst the discharge continued, the pain was rather alleviated; but it afterwards became worse than before, and, to use her own words, "the agony was now so great, that she would allow her arm to be cut off, rather than bear it any longer."

Every remedy having failed, acupuncturation was resolved upon. Accordingly, on the 13th, in the presence of Mr. Bacot, and gentlemen usually attending the Dispensary, Mr. David Duncan, an intelligent pupil, who had taken charge of the case from the beginning, introduced the needle at two different points in the upper and inner part of the forearm, for nearly three-fourths of an inch in depth. The same was done into the ball of the thumb, until the needle almost penetrated through the hand. The instrument was turned gently round at each introduction, but did not remain longer than fifteen seconds at a time. No blood followed, and the patient felt little pain in consequence, except when the needle was introduced into the muscles of the thumb.

For two days after, the pain in the forehead and thumb continued without any diminution, and it was now accompanied by a feeling of great numbness of the limb. On the third day, the pain was much alleviated, and the numbness had ceased. Next morning, she felt quite free from pain or any other complaint.

On the 9th of May, she came to the Dispensary, according to promise: she had suffered no return of pain, and the arm is as well, and feels even stronger, than before the first attack. A fortnight after this, she was visited at her own house: she still continued free from any recurrence of the disease.

*56, Grosvenor-street; June 1st, 1825.*

